# Using the Probability Density Function-Based Channel-Combination Bloch–Siegert Method Realizes Permittivity Imaging at 3T

**DOI:** 10.3390/bioengineering11070699

**Published:** 2024-07-10

**Authors:** Jiajia Wang, Yunyu Gao, Sherman Xuegang Xin

**Affiliations:** School of Biomedical Engineering, Southern Medical University, Guangzhou 510515, China

**Keywords:** electrical properties tomography, relative permittivity, ***B*_1_** mapping technology, Bloch–Siegert method

## Abstract

Magnetic resonance electrical properties tomography (MR EPT) can retrieve permittivity from the $B_{1}^{+}$ magnitude. However, the accuracy of the permittivity measurement using MR EPT is still not ideal due to the low signal-to-noise ratio (SNR) of $B_{1}^{+}$ magnitude. In this study, the probability density function (PDF)-based channel-combination Bloch–Siegert (BSS) method was firstly introduced to MR EPT for improving the accuracy of the permittivity measurement. MRI experiments were performed using a 3T scanner with an eight-channel receiver coil. The homogeneous water phantom was scanned for assessing the spatial distribution of $B_{1}^{+}$ magnitude obtained from the PDF-based channel-combination BSS method. Gadolinium (Gd) phantom and rats were scanned for assessing the feasibility of the PDF-based channel-combination BSS method in MR EPT. The Helmholtz-based EPT reconstruction algorithm was selected. For quantitative comparison, the permittivity measured by the open-ended coaxial probe method was considered as the ground-truth value. The accuracy of the permittivity measurement was estimated by the relative error between the reconstructed value and the ground-truth value. The reconstructed relative permittivity of Gd phantom was 52.413, while that of rat leg muscle was 54.053. The ground-truth values of relative permittivity of Gd phantom and rat leg muscle were 78.86 and 49.04, respectively. The relative error of average permittivity was 33.53% for Gd and 10.22% for rat leg muscle. The results indicated the high accuracy of the permittivity measurement using the PDF-based channel-combination BSS method in MR EPT. This improvement may promote the clinical application of MR EPT technology, such as in the early diagnosis of cancers.

## 1. Introduction

The electrical properties (EPs) of biological tissue mainly include conductivity and permittivity, which describe the properties of absorption and coupling electromagnetic energy in the electromagnetic field [1]. The EPs of biological tissues are determined by their own physiological characteristics, such as tissue water content, cell membrane structure, ion concentration, etc. [2]. Different tissues have different EPs [1]. For example, the conductivity and permittivity of muscle are 0.72 S/m and 63.45, respectively, at 128 MHz. For fat, they are 0.03 S/m and 5.92. The changes in tissues’ physiological and pathological states always raise the changes in EPs’ values [3,4]. Thus, the EPs value could reflect the physiological and pathological state of tissues, which is expected to be a biomarker in clinical diagnosis.

Noninvasive methods have been proposed for quantitatively imaging EPs, such as electrical impedance tomography (EIT) and magnetic resonance electrical impedance tomography (MR EIT). EIT is a noninvasive, radiation-free monitoring tool that allows real-time imaging [5,6,7]. However, estimation algorithms need to solve an ill-posed nonlinear problem. The solution may not be unique or may be extremely unstable. MR EIT measures EPs via probing the current distribution by magnetic resonance (MR) phase images. Comparing with EIT, MR EIT has high spatial resolution, but an electrical current still needs to be injected into the sample during MR scanning, which may cause safety concerns [8,9,10]; beyond that, the temporal resolution of MR EIT is relatively low [10]. The limitations of these methods hamper the clinical application of EPs.

Recently, an alternative approach, MR EPT, has been proposed, which is noninvasive, safe, has high resolution, and deviates the conductivity and the permittivity at the same time. Katscher et al. firstly established and verified the basic framework of MR EPT in 2009 [11]. The imaging process mainly consists of three steps: (a) MR signal acquisition, (b) $B_{1}^{+}$ field calculation, and (c) conductivity and permittivity reconstruction [11]. $B_{1}^{+}$ field calculation and EP reconstructions are the two core steps of MR EPT. Radio-frequency-transmitting field mapping technology, also known as $B_{1}$ mapping technology, is used to obtain the magnitude and phase of the complex $B_{1}$ field [12,13]. However, there is still a major challenge in directing the measurement of $B_{1}^{+}$ absolute phases. In this case, the $B_{1}^{+}$ phase and the $B_{1}^{+}$ magnitude are measured independently and differently. The phase maps are always retrieved with transceive phase approximation (TPA) [14]. Thus, its precision is linearly proportional to the SNR of the MR image. It is worth noting that in ultra-high-field (7T and above) MR systems, TPA is not directly applicable. On the other hand, $B_{1}^{+}$ magnitude is obtained by $B_{1}$ mapping methods. These methods utilize a model describing the sequence-specific $B_{1}^{+}$ encoding mechanism. This model regulates the noise propagation that leads to finite precision in the $B_{1}^{+}$ map. The mainstream $B_{1}$ mapping methods are actual flip-angle imaging (AFI) [15], BSS [16], and dual-refocusing echo acquisition mode (DREAM) [17] methods. A classical reconstruction strategy based on the Helmholtz equation uses the $B_{1}$ magnitude to reconstruct the permittivity and the $B_{1}$ phase to obtain conductivity [11,18,19]. The related research has been operated on different objects, such as the pelvis [20], brain [21,22], and breast [23]. The $B_{1}^{+}$ field was used as input data for the reconstruction algorithm, whose SNR directly impacts the reconstruction results [11]. Current research results indicated that reconstructions based on the phase of the $B_{1}^{+}$ field show better conductivity outcomes, whereas reconstructions based on the magnitude of the $B_{1}^{+}$ field for permittivity are unreliable. Analysis of the imaging principle revealed that reconstruction algorithms based on the Helmholtz equation involve second-order differences, making the permittivity imaging sensitive to noise [24,25]. Hence, a high SNR of $B_{1}^{+}$ magnitude is desired for improving the accuracy of permittivity imaging.

In order to obtain a high SNR $B_{1}^{+}$ magnitude for EPs’ reconstruction, several methods have been proposed to decrease the noise level. van Lier et al. incorporated linear smoothing filters into the discrete Laplacian operator [14], whereas Bulumulla et al. used skip factors in the discrete Laplacian operator [26]. However, adopting skip factors or using linear filters inevitably degrades the spatial resolution of the electrical property images [27]. Eric Michel et al. used an adaptive nonlinear filter to reduce the noise on the $B_{1}^{+}$ maps in 2014. However, using a smaller coil may complicate the EPT reconstructions because of the smaller phase delay and consequently the lower SNR of the $B_{1}^{+}\;$ maps. In 2018, Lei Guo et al. considered the influence of $B_{z}$ in EPs’ reconstruction, but the mutual coupling between the coil and the object was not considered [28]. Kyu-Jin Jung et al. denoised the $B_{1}^{+}$ phase using a deep learning method for phase-based in vivo electrical conductivity reconstruction in a 3T MR system. Data acquired with the transceive assumption was invalid at ultra-high-field strengths [18]. Soraya Gavazzi et al. investigated the sequence-specific impact of $B_{1}^{+}$ magnitude mapping on the accuracy and precision of permittivity reconstruction using 3T in the pelvic region [29]. Average permittivity bias relative to the true permittivity was 7% to 20% for AFI and BSS and 12% to 35% for DREAM. BSS demonstrated good imaging results. However, in vivo MR measurements of the permittivity imaging did not provide reliable quantitative estimation of permittivity imaging. Sharma adopted the weighted-average BSS method to combine channels according to the image amplitude of each receiving channel [30]. The WA BSS method does not consider the effect of the noise of the phase image, which could lead to large errors at high noise levels. However, the existing research works still have significant errors in the practical imaging of permittivity, indicating a considerable gap for clinical application. In our previous work, we proposed the PDF-based channel-combination BSS method for obtaining the $B_{1}^{+}$ magnitude, which is effective and insensitive to noise. BSS methods are always combined with a multi-channel array coil, which has the advantages of high sensitivity, high SNR, and high spatial resolution of images [31,32], and is usually used as the receiving coil during MR scanning [33]. Each channel simultaneously collects signals independently. The PDF-based channel-combination BSS method assumes that the signal phase difference of the MR signal of each receiving channel is expressed as a probability density function. By using specific computational methods, the SNR of $B_{1}$ can be further enhanced [34]. However, the feasibility of the PDF-based channel-combination BSS method in permittivity imaging still remains to be validated.

In this study, the PDF-based channel-combination BSS method was utilized to improve the accuracy of the $B_{1}^{+}$ magnitude, ultimately enhancing the imaging accuracy of the permittivity in in vivo MR experiments. The confirmatory experiment of the $B_{1}$ field was carried out at 3T. Gd phantom and rat experiments were conducted to evaluate the performance of the method. The permittivity reconstructed results were compared with the ground-truth values, measured by the open-ended coaxial probe method, which showed good consistency between the two methods. The study is expected to advance the application of permittivity measurements in the clinic.

## 2. Theory and Methods

**Figure 1:**
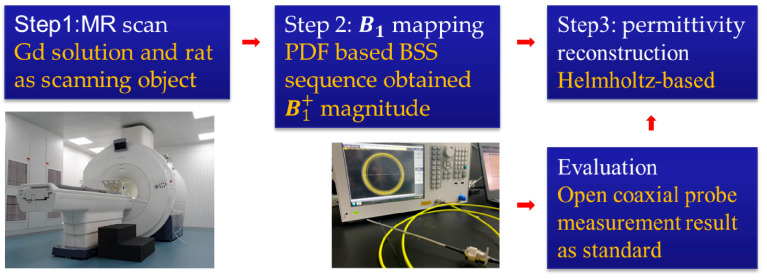
Scheme of the PDF-based channel-combination BSS for MR EPT, consisting of MR signal acquisition, the $B_{1}$ mapping technique, and permittivity reconstruction. Firstly, the MR image was obtained using the MR scanner. Then, the PDF-based channel-combination BSS sequence was performed to retrieve the $B_{1}^{+}$ magnitude needed for permittivity reconstruction. After that, the Helmholtz-based EPT reconstruction method was used to image the permittivity maps. Finally, the permittivity, measured by the open-ended coaxial probe method, was used as the ground-truth value to validate the accuracy of the EPT reconstructed results.

This study involves the $B_{1}$ mapping technique, MR EPT imaging technique, and open-ended coaxial probe measurement technique. Figure 1 shows the scheme of the PDF-based channel-combination BSS MR EPT. The first step was to collect magnetic resonance signals through magnetic resonance. The Gd phantom and rats were chosen for scanning. The second step was obtaining the $B_{1}^{+}$ magnitude used for permittivity reconstruction. At this step, the PDF-based BSS method was selected as the $B_{1}$ mapping technique. Then, the Helmholtz-based EPT reconstruction method was used to image the permittivity maps based on the $B_{1}^{+}$ magnitude. Subsequently, the accuracy was evaluated by calculating the relative error between the reconstructed values of permittivity and the ground-truth measured by the open-ended coaxial probe measurement technique. 

### 2.1. BSS Method

The BSS method is one of the $B_{1}$ mapping technologies based on the phase of the MR signal. It applies a strong off-resonance pulse immediately after excitation, resulting in a phase shift. This methods applies two scans with symmetric offset frequencies during the MR scanning. 

The magnitude of the $B_{1}^{+}$ field could be derived as:

(1) $$\;B_{1}=\sqrt{\frac{{\Delta \phi }_{\;}^{\;}}{2K_{BS}}}$$

where $K_{BS}=\int_{0}^{\tau }\frac{{\left(\gamma B_{norm}\left(t\right)\right)}^{2}}{2\Delta \omega }dt$, ***B***_norm_(t) is the detuned pulse, Δω is the off-resonance pulse, and τ is the duration of the off-resonance pulse.

### 2.2. PDF-Based Channel-Combination BSS Method

The multi-channel coil is one of the commonly used receiving coils in magnetic resonance scanning. Each channel can simultaneously collect signals independently. When using the $B_{1}$ mapping technique to determine the $B_{1}$ magnitude, the signals of each channel need to be combined. The common method is to square and sum the signals of each channel. In this paper, the PDF-based channel-combination BSS was used to solve the $B_{1}$ magnitude. The signal phase difference of the MR signal of each receiving channel was expressed as a probability density function. The signals collected by each receiving channel contained noise, regarded as independent samples, and the maximum likelihood method was adopted to find the unbiased estimate of the signal phase difference in the MR signals of each channel [34]. Compared to the WA method, the PDF-based channel-combination BSS improved the accuracy of the $B_{1}^{+}$ magnitude by enhancing the robustness against noise [34]. Moreover, $\left|B_{1}^{+}\right|$, as the data source of relative permittivity reconstruction, could improve the permittivity reconstruction accuracy. 

Two scanning signals were acquired. The phase differences of the channel coils were determined as follows:

(2) $$\phi_{i}^{+}(r)={\bar{\phi }}_{B_{1}^{+}}^{+}(r)+\phi_{i,common}^{\;}(r)+\varphi_{i}^{+}(r)$$

(3) $$\phi_{i}^{-}(r)={\bar{\phi }}_{B_{1}^{+}}^{-}(r)+\phi_{i,common}^{\;}(r)+\varphi_{i}^{-}(r)$$

where i = 1, 2, …, n, indicates the i-th channel, and $\phi_{i}^{\;}$ indicates the signal phase of the i-th channel. The superscript + represents the signal collected in the first scan, and the superscript represents the signal collected in the second scan. ***r*** stands for spatial position, which will be omitted later. ${\bar{\phi }}_{B_{1}^{+}}^{-}$ denotes the $\left|B_{1}^{+}\right|$ related to the size of the phase. $\phi_{i,common}^{\;}$ represents the background phase, which is independent of the $\left|B_{1}^{+}\right|$, and $\varphi_{i}^{\;}$ represents signal phase noise.

Equation (2) minus Equation (3) yields:

(4) $${\Delta \phi }_{i}^{\;}=\phi_{i}^{+}-\phi_{i}^{-}={\bar{\phi }}_{B_{1}^{+}}^{+}-{\bar{\phi }}_{B_{1}^{+}}^{-}+\left(\varphi_{i}^{+}-\varphi_{i}^{-}\right)$$

where $\varphi_{i}^{+}$ and $\varphi_{i}^{-}$ are the noise of the phase. ${\Delta \phi }_{i}$ obeys the Gaussian distribution, assuming that $\Delta {\bar{\phi }}_{\left|B_{1}^{+}\right|}^{\;}={\bar{\phi }}_{B_{1}^{+}}^{+}-{\bar{\phi }}_{B_{1}^{+}}^{-}$ is the mean value of ${\Delta \phi }_{i}^{\;}$ related to the $B_{1}^{+}$ magnitude. 

Assuming that each receiving channel noise is independent of each other, the probability density function of the signal phase difference, ${\Delta \phi }_{i}^{\;}$, can be expressed as:

(5) $$P_{{\Delta \phi }_{i}^{\;}}\left({\Delta \phi }_{i}^{\;}\right)=\frac{1}{\sqrt{4\pi {\left(\sigma_{i}/A_{i}\right)}^{2}}}exp\left(-\frac{{\left({\Delta \phi }_{i}^{\;}-\Delta {\bar{\phi }}_{\left|B_{1}^{+}\right|}^{\;}\right)}^{2}}{4{\left(\sigma_{i}/A_{i}\right)}^{2}}\right)$$

The maximum likelihood is utilized to estimate $\Delta {\bar{\phi }}_{\left|B_{1}^{+}\right|}^{\;}$, as follows:

(6) $$L\left(\Delta {\bar{\phi }}_{\left|B_{1}^{+}\right|}^{\;}\right)=\ln\left[\prod_{i=1}^{n}P_{{\Delta \phi }_{i}^{\;}}\left({\Delta \phi }_{i}^{\;}\right)\right]=\sum_{i=1}^{n}\ln P_{{\Delta \phi }_{i}^{\;}}\left({\Delta \phi }_{i}^{\;}\right)$$

Substituting Equation (5) into Equation (6) yields:

(7) $$L\left(\Delta {\bar{\phi }}_{\left|B_{1}^{+}\right|}^{\;}\right)=-\frac{n}{2}\ln\left(2\pi \right)-\sum_{i=1}^{n}\left(\ln\left(2(\sigma_{i}/A_{i})\right)+\frac{{\left({\Delta \phi }_{i}^{\;}-\Delta {\bar{\phi }}_{\left|B_{1}^{+}\right|}^{\;}\right)}^{2}}{4{\left(\sigma_{i}/A_{i}\right)}^{2}}\right)$$

As for $dL\left(\Delta {\bar{\phi }}_{\left|B_{1}^{+}\right|}^{\;}\right)/d\Delta {\bar{\phi }}_{\left|B_{1}^{+}\right|}^{\;}=0$, the maximum likelihood function (6) takes the maximum, and the unbiased estimation of $\Delta {\bar{\phi }}_{\left|B_{1}^{+}\right|}^{\;}$ was obtained.

### 2.3. Helmholtz-Based EPT

The Helmholtz-based EPT imaging process can be broken down into three main steps: MR signal acquisition, $\left|B_{1}^{+}\right|\;$ mapping method, and EPs’ reconstruction. The scheme of the Helmholtz-based MR EPT is shown in Figure 1.

From Maxwell’s equation:

(8) ∇×E=−iωB

(9) $$\nabla \times B=i\omega \mu_{0}\gamma E$$

Equation (8) is Faraday’s law of electromagnetic induction and Equation (9) is the ampere circuit law, where E is the electric field intensity, B is the magnetic field intensity, ω is the Larmor frequency, $\mu_{0}$ is the permeability of the vacuum, and the value is $4\pi \times 10^{-7}$ H/m, and γ is the complex dielectric constant:

(10) $$\gamma =\varepsilon_{0}\varepsilon_{r}-i\frac{\sigma }{\omega }$$

where $\varepsilon_{0}$ is the permittivity of the vacuum, $\varepsilon_{r}$ is the relative permittivity, and σ is the conductivity.

Considering ∇γ=0 and $B_{z}=0$, according to the electromagnetic reciprocity principle $B_{1}^{+}=\frac{1}{2}{(B}_{x}+iB_{y})$, the reconstruction equation could be written as:

(11) $$\nabla^{2}B_{1}^{+}+\omega^{2}\mu_{0}\gamma B_{1}^{+}=0$$

Separating the real part and the imaginary part, the calculation formula of relative permittivity could be derived as:

(12) $$\varepsilon_{r}=\frac{-1}{\omega^{2}\mu_{0}\varepsilon_{0}}Re\left(\frac{\nabla^{2}B_{1}^{+}}{B_{1}^{+}}\right)$$

### 2.4. Open-Ended Coaxial Probe Method

The measurement method of the open-ended coaxial probe is based on the transmission line theory. When the coaxial line is in contact with the sample to be measured, the impedance between the probe terminal and the sample appears as a mismatch. The electromagnetic waves are emitted at the probe terminal. The measurement signal is generated by the network analyzer, and the reflected signal with the electrical property information of the sample is post-processed. Then, the reflection coefficient is obtained. The network analyzer captures the reflected signal and calculates the dielectric property value of the tissue based on the reflected signal [35]. The open-ended coaxial probe method has simple measuring equipment, a wide measuring frequency band, and is suitable for measuring objects in different physical states, such as liquid, semi-solid, and solid, which is very suitable for the measurement of biological tissues [35]. The open-ended coaxial probe measurement system consists of a network analyzer, coaxial line, and a laptop computer. The EPs measured by the open-ended coaxial probe can be used as the gold standard for the EP value of MR EPT.

The proposed calibration method consists of calculating three parameters, $A_{1}$, $A_{2}$, and $A_{3}$, as follows:

(13) $$A_{1}=\frac{{(\rho }_{2}-\rho_{1})+{(\rho }_{1}-\rho_{3})(\varepsilon_{0}\varepsilon_{r}-j\frac{\sigma }{\omega })}{\rho_{3}-\rho_{2}}$$

(14) $$A_{2}=\frac{{\rho_{3}(\rho }_{2}-\rho_{1})+{\rho_{2}(\rho }_{1}-\rho_{3})(\varepsilon_{0}\varepsilon_{r}-j\frac{\sigma }{\omega })}{\rho_{3}-\rho_{2}}$$

(15) $$A_{3}=\rho_{1}$$

where $\rho_{1}$, $\rho_{2}$, and $\rho_{3}$ are the reflection coefficients with three standard terminations: the short-circuit, open-circuit, and loaded-circuit, respectively.

(16) $$\gamma =\varepsilon_{0}\varepsilon_{r}-i\frac{\sigma }{\omega }=\frac{A_{1}\rho_{m}-A_{2}}{A_{3}-\rho_{m}}$$

where $\rho_{m}$ is the reflection coefficient of the calibration surface measured by the network analyzer.

The relative permittivity measured by the open-ended coaxial probe can be calculated by the real part of Equation (17):

(17) $$\varepsilon_{r}=RE\left[\frac{A_{1}\rho_{m}-A_{2}}{A_{3}-\rho_{m}}\right]$$

### 2.5. Accuracy Evaluation of Imaging Results

We calculated the relative error to evaluate the accuracy of the reconstructed results and the ground-truth value measured by the open-ended coaxial probe:

(18) $${Re}_{\varepsilon_{r}}=\frac{\varepsilon_{rRec}-\varepsilon_{rMeas}}{\varepsilon_{rMeas}}$$

where $\varepsilon_{rMeas}$ represents the true value of the relative permittivity and $\varepsilon_{rRec}$ is the reconstruction value. 

### 2.6. MRI Experiments

We used common experimental designs in the field of MR EPT. We selected Ga phantoms to represent homogeneous subjects and rats to represent inhomogeneous subjects as the imaging objects. The imaging experiments were conducted multiple times.

#### 2.6.1. Phantom Experiment

A 0.1 mol/L Gd solution was placed in a plastic water bottle to serve as an imaging model for magnetic resonance imaging using the 3T MR System (Basda Medical, Shenzhen, China). The volume coil was used to transmit the radio frequency, and the array coil of 8 channels was used to receive the signal. The scanning sequence used was the BSS sequence.

#### 2.6.2. In Vivo Rat Experiment

An in vivo experiment was performed on Wista rats using a 3T MR scanner. The volume coil served as the transmitting coil, while the 8-channel coil acted as the receiving coil. Fermi pulses with a ±4 kHz frequency offset were employed to encode $B_{1}^{+}$ into signal phases. The acquisition parameters are as follows: TE = 10 ms, TR = 633 ms, FOV = 128 × 128 mm^2^, matrix size = 256 × 180, slice thickness = 2 mm, and number of slices = 18. The rats underwent gas anesthesia to maintain their position stability during MRI scanning. The scanning time of the BSS sequence was approximately 40 min. The concentration of isoflurane released by the gas anesthesia machine was controlled within the appropriate range, simultaneously warming the rat.

## 3. Results

**Figure 2:**
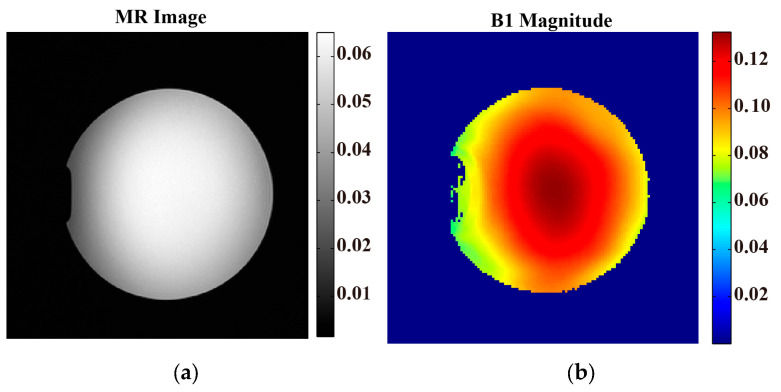
Verification of the PDF-based BSS method. The MR image (**a**) of the uniform water phantom was obtained by the 3T MR scanner. (**b**) The magnitude of $B_{1}^{+}$. The MR images show a uniform distribution of the Gd phantom, representing the imaging characteristics of a homogeneous body model. The amplitude of the $B_{1}^{+}$ magnitude gradually increased from the periphery toward the center.

**Figure 3:**
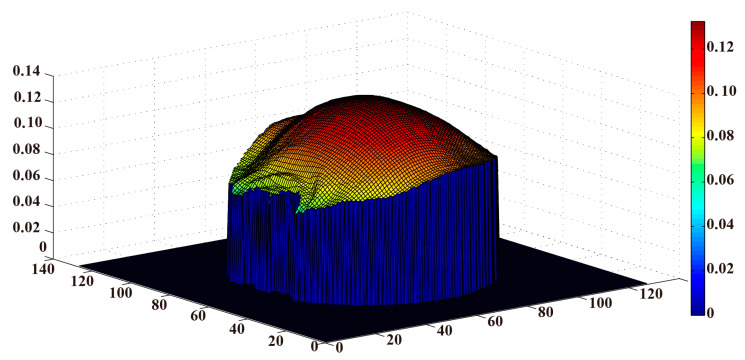
Three-dimensional view of the $B_{1}^{+}$ magnitude. The $B_{1}^{+}$ magnitude smoothly increased from the periphery toward the center. The maximum of $\left|B_{1}^{+}\right|\;$ was 0.1325.

Figure 2 illustrates the feasibility of $\left|B_{1}^{+}\right|$ obtained by the PDF-based channel-combination BSS method via the multi-channel coil. Figure 2a displays the MR image of the Gd phantom. The MR images showed a uniform distribution of the Gd phantom, representing the imaging characteristics of a homogeneous body model. Figure 2b shows that $\left|B_{1}^{+}\right|$ had a good distribution on spatial continuity, and the $B_{1}^{+}$ magnitude gradually increased from the periphery toward the center. It is important to note that the $B_{1}^{+}$ magnitude increased smoothly, as shown in Figure 3. The maximum of $\left|B_{1}^{+}\right|\;$ was 0.1325. The distribution of $\left|B_{1}^{+}\right|$ with high SNR was the basis of the permittivity reconstruction.

**Figure 4:**
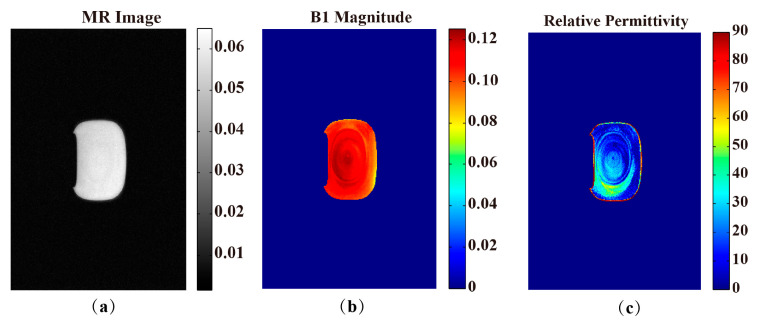
Results of the Gd phantom imaging using 3T, including MR image (**a**); magnitude of the $B_{1}^{+}$ field (**b**); the reconstructed result of relative permittivity (**c**). Every three ROI values were chosen to calculate the permittivity. After that, we obtained the average permittivity of the entire ROI as 52.4130 ± 1.9188.

Figure 4 displays the results of the Gd phantom imaging experiment using 3T. The 5th slice was chosen to be shown. Each slice included the MR image, $B_{1}^{+}$ magnitude, and permittivity. Firstly, we obtained the Gd phantom MR image in Figure 4a. Then, we used the PDF-based channel-combination method to obtain the $B_{1}^{+}$ magnitude in Figure 4b. Figure 4c shows the reconstructed value of relative permittivity. Figure S1 in Supplementary Materials shows the imaging results of the Gd phantom and water phantom for supporting this conclusion. Figure S2 in Supplementary Materials presents a picture of the Gd phantom and water phantom.

**Figure 5:**
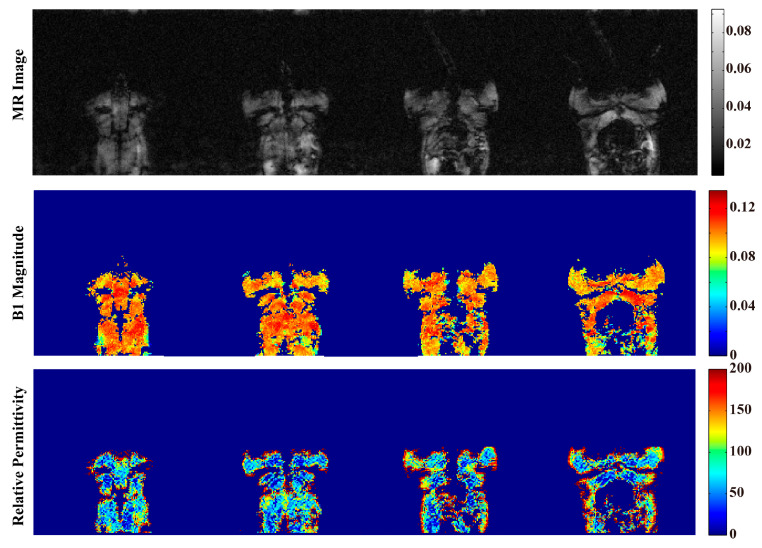
Results of the rat imaging experiment at 3T. The MR image is displayed on the 1st row, and the magnitude of the $B_{1}^{+}$ field is shown in the 2nd row. The 3rd row is the reconstructed value of relative permittivity. The average permittivity of each slice was calculated. On this basis, we further calculated the average relative permittivity across all levels. The average value of the relative permittivity of rat muscles was 54.0530  ± 4.0269.

Figure 5 exhibits the rat in vivo experiment results at 3T. Four slices are shown. The first row contains the MR image, the second row includes the magnitude of the $B_{1}^{+}$ field, and the third row displays the relative permittivity value. The average permittivity of each slice was calculated. On this basis, we further calculated the average relative permittivity across all levels. The reconstructed value of the relative permittivity of the rat leg muscles was 54.0530  ± 4.0269. 

**Figure 6:**
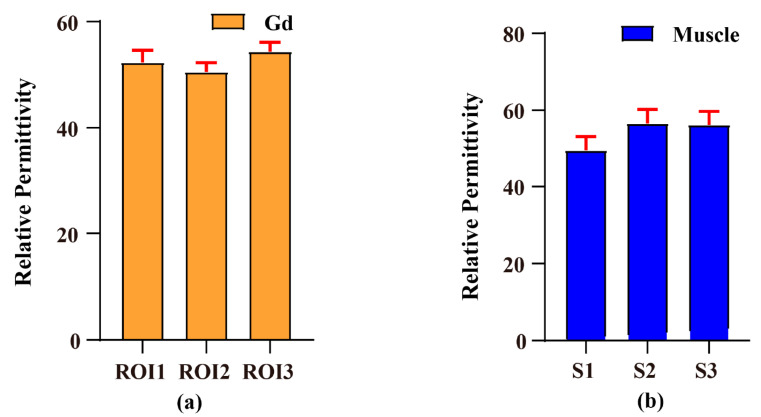
Results of relative permittivity. (**a**) The ROI permittivity of the Gd phantom. (**b**) The permittivity from the 4th slice to the 6th slice of the rat leg muscle. The ‘mean ± SD’ values of the permittivity of each ROI were 52.3078 ± 2.0386, 50.5489 ± 1.7194, and 54.3822 ± 1.7154 (**a**). The ‘mean ± SD’ values of permittivity from slice 4 to slice 6 were 49.4100 ± 12.9257, 56.5933 ± 13.1388, and 56.1556 ± 12.5540, respectively, as shown in (**b**).

Figure 6 illustrates the results relative permittivity of the Gd phantom and the rat leg muscle. The distribution of relative permittivity was not uniform. We chose the region of interest (ROI) to calculate the average permittivity. The ‘mean ± SD’ values of permittivity of each ROI were 52.3078 ± 2.0386, 50.5489 ± 1.7194, and 54.3822 ± 1.7154, as shown in Figure 6a. The average value of the entire phantom was 52.4130 ± 1.9188. Three slices of permittivity images of the rat leg muscle were chosen to calculate the average permittivity of the rat leg muscles. The ‘mean ± SD’ values of permittivity from slice 4 to slice 6 were 49.4100 ± 12.9257, 56.5933 ± 13.1388, and 56.1556 ± 12.5540, respectively, as shown in Figure 6b. The imaging accuracy significantly improved in in vivo imaging compared to existing research results, especially in the global imaging results [27,29]. Then, we calculated the average value of all the slices. 

**Figure 7:**
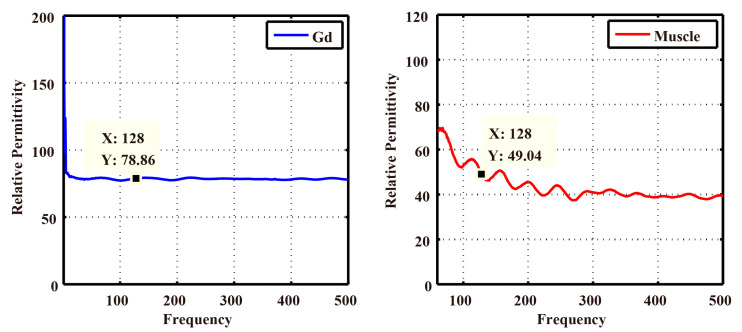
Relative permittivity of Gd phantom and rat muscles measured by the open-ended coaxial probe at 0–500 MHz. The x-axis represents the frequency, and the y-axis is the value of the relative permittivity. The blue line shows the relative permittivity of Gd at the frequency from 0 to 500 MHz and the red line displays the relative permittivity of muscle. The two black points on the blue and red lines point out that the relative permittivity was 78.86 of Gd and 49.04 of muscle at a frequency of 128 MHz.

The results of the open-ended coaxial probe measurements on Gd phantom and rat leg muscles in the frequency range of 0–500 MHz are shown in Figure 7. The relative permittivity values were 78.86 for Gd and 49.04 for muscle, which served as reference values for the EPT relative permittivity imaging results. Table 1 displays the comparison between the EPT reconstructed values and the probe measurements, showing the relative errors of 33.53% for Gd and 10.22% for muscle.

## 4. Discussion

This work investigated the feasibility of the PDF-based multi-channel combination BSS method to reconstruct the relative permittivity of the phantom and the rat muscles, and the results indicated that the PDF-based multi-channel combination BSS method could improve the accuracy of the permittivity. At present, the imaging research on electrical properties mainly focuses on conductivity in comparison to relative permittivity. The reason is that the relative permittivity reconstruction algorithm based on $B_{1}$ magnitude is more susceptible to noise. Therefore, it is vitally important to select an appropriate $B_{1}^{+}$ mapping sequence, which could obtain a $B_{1}^{+}$ magnitude map with a high SNR.

It should be noted that the framework shown in Figure 1 is also suitable for all $B_{1}^{+}$ mapping sequences. The previous study indicated that the BSS sequence could always obtain the highest SNR among commonly available sequences [29]. For example, the maximum $B_{1}^{+}$ bias relative to the true $B_{1}^{+}$ distribution was 1% for BSS and 6% to 15% for DREAM. Therefore, only the BSS sequence was adapted in our study.

In this study, the PDF-based BSS method was selected to obtain the $B_{1}^{+}$ magnitude map for relative permittivity imaging. The feasibility of PDF-based BSS sequence imaging was firstly validated. As shown in Figure 3, the three-dimensional view clearly displays the variation tendency of the $B_{1}^{+}$ magnitude. The $B_{1}^{+}$ magnitude gradually increased from the sides to the center. The continuity of distribution well meets the needs of the RF field [34]. Thus, the feasibility of the multi-channel combination BSS method was verified.

Compared with previous studies, the quality of the relative permittivity map of our study was better. In our phantom experiment, as shown in Figure 4c, the permittivity value of the Gd phantom was more homogeneous compared with other phantom studies [27]. In our rat experiment, as shown in the 3rd row of Figure 5, the contrast of different tissues was significantly improved compared with in vivo experiment results in other studies [29].

To make a quantitative comparison with other studies, we calculated the relative error of relative permittivity. In our study, the relative error of relative permittivity in rat leg muscle was 10.22%, whereas the relative error of relative permittivity measured using the BSS method was 20% in [29]. This demonstrated that the PDF-based BSS method has a higher measurement accuracy than the BSS method.

The PDF-based BSS combination method is based on an assumption that the biological tissue is homogeneous. The relative error of permittivity not only depends on the reconstruction method, but also on tissue geometry. Thus, in the rat experiment, we chose the relatively homogeneous and large rat leg muscle rather than the smaller tissue or the intersection of the tissue for comparison and analysis. 

The open-ended coaxial probe method [36] is regarded as the gold standard to evaluate the accuracy of the permittivity measurement in the MR EPT field [37], and it has been used to build up the EP databases of biological tissues [38,39,40]. Therefore, in our study, the open-ended coaxial probe method was selected as the validation method, and the relative error of the calculated permittivity of Gd phantom and rat leg muscles show in Table 1.

Although the proposed method in this study obtained a high-precision $B_{1}^{+}$ magnitude measurement, there are still methodlogical limitations. First, it is based on the unbiased estimation of signals from each receiving channel. The $B_{1}^{+}$ magnitude obtained by this method was still accompanied with noise [34]. Therefore, the reconstructed relative permittivity still exhibited reconstruction errors. In addition, the precision of the $B_{1}^{+}$ magnitude measurement was linearly proportional to the signal-to-noise ratio (SNR) of the MR image [41]. Thus, the factors influencing the SNR of the MR image also decreased the SNR of the $B_{1}^{+}$ magnitude map, such as the inhomoginety of the $B_{0}$ field. Our study did not comprehensively consider the influence of all factors. In future studies, in order to obtain reliable and high-quality MR images as much as possible, it is important to understand the correlation between various factors and MR image quality, and carefully consider these factors during the experiments.

## 5. Conclusions

In this paper, the PDF-based multi-channel combination BSS method combined with the Helmholtz reconstruction algorithm was used to reconstruct the permittivity. The feasibility of the PDF-based multi-channel combination BSS method was firstly verified by a water model. The method was validated by Gd phantom and rat muscle experiments. Results of reconstructed permittivity were compared with those measured by the open-ended coaxial probe. The results of these two methods showed good agreement. Compared with the existing methods, the PDF-based multi-channel combination BSS method was utilized to improve the accuracy of the $B_{1}^{+}$ magnitude, ultimately enhancing the imaging accuracy of the in vivo permittivity imaging, which promotes the clinical application of relative permittivity. However, the accuracy of the objects with an inhomogeneous structure still needs to be further improved at the boundary.

## Figures and Tables

**Table 1:** Relative error of calculated permittivity of Gd phantom and rat leg muscles.

Method/Subjects	PDF-Based BSS Combination EPT	Open-Ended Coaxial Probe	Relative Error (%)
Gd phantom	52.413 ± 1.919	78.86	33.53
Rat leg muscles	54.053 ± 4.027	49.04	10.22

## Data Availability

The datasets generated during the current study are available from the corresponding author upon a reasonable request.

## Supplementary Materials

The following supporting information can be downloaded at: https://www.mdpi.com/article/10.3390/bioengineering11070699/s1, Figure S1: Results of the Gd phantom imaging and water phantom at 3T. (a) is MR image, (b) shows the magnitude of B_1^+ field; (c) is the reconstructed result of relative permittivity. from the relative permittivity map. Three ROIS were chosen to caculate the permittivity. Figure S2: Scanned object. The bottom is a bag with water, and the top is a bottle with 0.1 mol/L gadolinium solution.
