# Supplementary material for: Using the Probability Density Function-Based Channel-Combination Bloch–Siegert Method Realizes Permittivity Imaging at 3T
Source: Bioengineering (Basel). 2024 Jul 10;11(7):699. doi: 10.3390/bioengineering11070699 (PMC11274052; doi:10.3390/bioengineering11070699)
Supplement: Supplementary file 1 [file bioengineering-11-00699-s001.zip › bioengineering-3048888-supplementary.pdf]

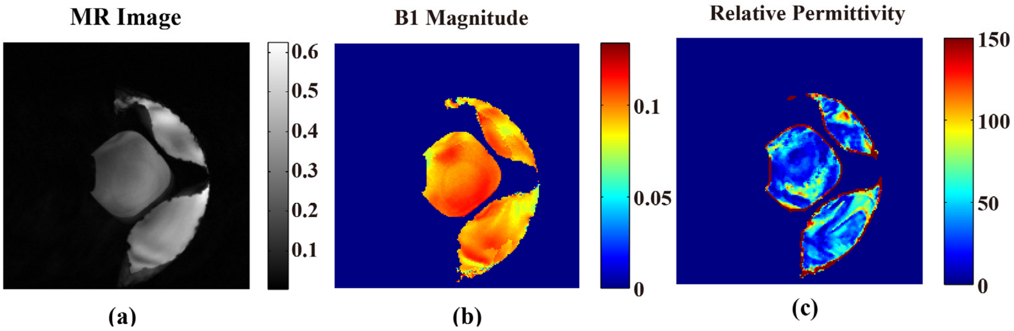

Figure S1. Results of the Gd phantom imaging and water phantom at 3T. (**a**) is MR image, (**b**) shows the magnitude of $\boldsymbol{B}_1^+$ field; (**c**) is the reconstructed result of relative permittivity. from the relative permittivity map. Three ROIS were chosen to caculate the permittivity.

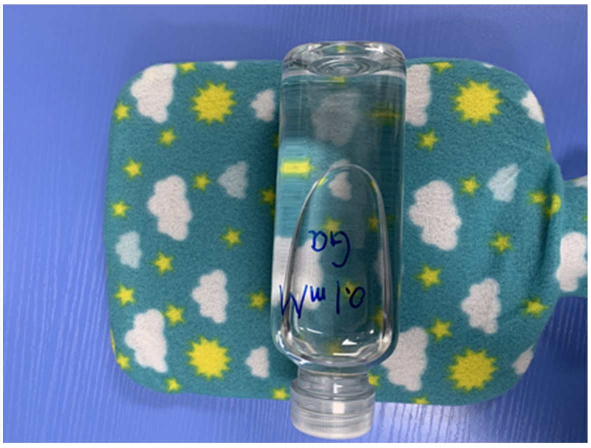

Figure S2. Scanned object. The bottom is a bag with water, and the top is a bottle with 0.1 mol/L gadolinium solution
